# A mixed solution-processed gate dielectric for zinc-tin oxide thin-film transistor and its MIS capacitance

**DOI:** 10.1038/srep33576

**Published:** 2016-09-19

**Authors:** Hunho Kim, Young-Jin Kwack, Eui-Jung Yun, Woon-Seop Choi

**Affiliations:** 1 Department of Display Engineering, Hoseo University Asan, 31499, Korea; 2 Department of ICT and Automotive Engineering, Hoseo University Asan, 31499, Korea

## Abstract

Solution-processed gate dielectrics were fabricated with the combined ZrO_2_ and Al_2_O_3_ (ZAO) in the form of mixed and stacked types for oxide thin film transistors (TFTs). ZAO thin films prepared with double coatings for solid gate dielectrics were characterized by analytical tools. For the first time, the capacitance of the oxide semiconductor was extracted from the capacitance-voltage properties of the zinc-tin oxide (ZTO) TFTs with the combined ZAO dielectrics by using the proposed metal-insulator-semiconductor (MIS) structure model. The capacitance evolution of the semiconductor from the TFT model structure described well the threshold voltage shift observed in the ZTO TFT with the ZAO (1:2) gate dielectric. The electrical properties of the ZTO TFT with a ZAO (1:2) gate dielectric showed low voltage driving with a field effect mobility of 37.01 cm^2^/Vs, a threshold voltage of 2.00 V, an on-to-off current ratio of 1.46 × 10^5^, and a subthreshold slope of 0.10 V/dec.

Amorphous metal-oxide thin-film transistors have attracted considerable interest as the backplane electronics for active matrix organic light emitting diodes (AMOLED) and transparent displays because of their outstanding properties such as high optical transparency, high mobility, good compatibility, and low temperature processability compared to commercial amorphous silicon TFTs. Among the many semiconductors in oxide TFTs, ZTO has drawn attention because it does not contain expensive rare elements, such as indium and gallium. Because ZnO based TFTs easily produce oxygen vacancies and carrier traps in the vacancies, many different approaches for the development of ZTO TFTs with high performance have been incorporated such as precursors, compositions, and various annealing processes^1^,^2^,^3^. The optimized properties of the oxide semiconductor and gate dielectric are crucial for the better TFT performance. Power consumption is a key issue for mobile electronics applications due to the limited capacity of the rechargeable battery^4^. The high k-dielectric as a gate insulator is attractive because of the capacitive coupling and reduced power consumption^4^. This can provide high capacitance with a very thin layer as well as increase the driving current and lower the operating voltage^5^. The high capacitance with a thicker layer will allow effective charge injection and reduce the leakage current^6^. Among them, zirconium oxide (ZrO_2_)^7^, aluminum oxide (Al_2_O_3_)^8^ and hafnium oxide (HfO_2_)^9^ have been studied. Recently, solution-processed high-k gate dielectrics have attracted much attention due to printing capability, large area process, low cost device, and compatibility with flexible substrates. These inorganic dielectrics are more suitable for low driving oxide TFTs than organic dielectrics due to their large conduction band offset and excellent solvent resistance^10^. Al_2_O_3_ films are well known high-k dielectrics because of their interfacial trap density with oxide semiconductors and the relative permittivity of 9^11^. ZrO_2_ is also a promising material because of its high permittivity, wide band gap, and thermal stability^4^. Recently, Hasan *et al*. prepared a solution-processed ZTO/ZrO_2_ dielectric and showed a mobility of 53 cm^2^/Vs with a 1 V operating voltage^12^. Branquinho reported the ZTO/Al_2_O_3_ dielectric showing 0.8 cm^2^/Vs with a 4 V operation^13^. Gao fabricated a solution-processed Al-doped ZrO_2_ gate dielectric for oxide TFTs, showing 19.67 cm^2^/Vs^14^. Jo *et al*. produced IGZO TFTs using a Zr doped Al_2_O_3_ gate dielectric with UV annealing, revealing a mobility of 20–40 cm^2^/Vs with an excellent stability^15^. Kim *et al*. reported high-k Hf and Al oxide as a stacked gate dielectric for ZTO TFTs, showing better properties with Al_2_O_3_ on the HfO_2_ structure^16^.

On the other hand, there are few reports on two-mixed solution-processed gate dielectrics on oxide TFTs. Therefore, it is important to investigate the gate dielectrics, as a mixed single layer and stacked double layer structures, composed of a highly insulating aluminum oxide and a highly polarizable zirconium oxide with strong bonding ability to oxygen. Thin films and capacitance-voltage properties of the solution-processed and combined dielectric with ZrO_2_ and Al_2_O_3_ (ZAO) dielectric were characterized thoroughly by analytical methods. The electrical properties with a low voltage driving of ZTO TFTs prepared by various solution-processed ZAO gate dielectrics were evaluated. The capacitance relationship between the dielectrics and active layer was proposed for the first time using metal-insulator-semiconductor (MIS) structure model.

## Results and Discussion

**Figure 1:**
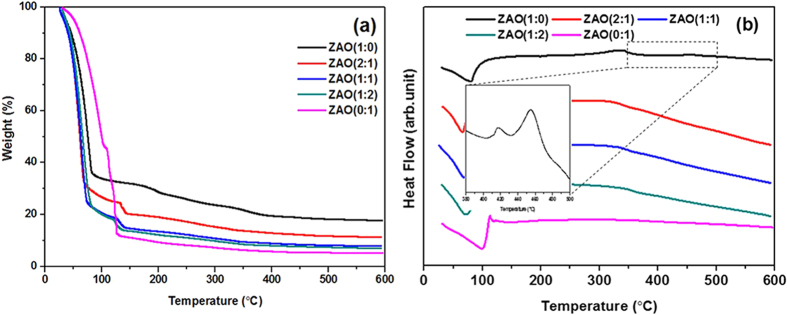
(**a**) TGA and (**b**) DSC thermograms of ZAO solution with a Zr:Al mole ratio of 1:0, 2:1, 1:1, 1:2, and 1:0 measured under N_2_.

Thermal analyses of the ZAO dried films with various mole ratios were performed by TGA/DSC after solvent evaporation at 70 °C for 20 h as shown in Fig. 1. A weight loss of more than 50% was observed at 150 °C, which was attributed to the solvent evaporation, such as 2-methoxyethanol, the decomposition of the organic group-associated metal salts^17^, and the hydrolysis of the ZrO_2_ solution from zircony chloride octahydrate to zirconyl hydroxylchloride^18^. Most samples except for ZAO (0:1, aluminum only) showed a weight loss at approximately 250–350 °C due to the dihydroxylation behavior of the zirconium precursor^19^. The broad exothermic peak approximately 340 °C in Fig. 1(b) indicates gradual densification, which forms a metal-oxygen frame work, to make dense films by decreasing the impurities in the films^18^. The ZAO (1:0, zirconium only) sample showed an exothermic peak at 417 and 457 °C due to the crystallization of zirconium^18^ and the similar temperature of the zirconium chloride melting point. ZAO dielectric thin films from some Zr:Al mole ratios of 2:1, 1:1, and 1:2 did not show a crystallization peak because aluminum addition to zirconium disrupts the crystallization behavior and decreases the dihydroxylation temperature^19^. The addition of Al_2_O_3_ to the ZrO_2_ matrix affects the nucleation and growth of ZrO_2_ in the crystalline phase^20^. Above 450 °C, all mixtures of precursors turn to the metal-oxide form after complete removal of the solvent and organic residues.

XRD analyses of the ZAO gate dielectric thin film are shown in Fig. S1 in the Supplementary Information (SI). The ZrO_2_ and Al_2_O_3_ thin films remained amorphous less than 400 °C and at 550 °C, respectively^18^,^21^. The ZAO (1:0, ZrO_2_ only) thin films showed the crystalline (111) peak at 30° 2θ°. While the rest of ZAO thin films did not exhibit a crystalline peak^18^. The metal oxide can be formed from a metal hydroxide via a condensation reaction between the adjoining hydroxyl groups with the removal of water. A continuous condensation reaction leads to an extended network of metal oxygen metal (M-O-M) bonds, which in turn leads to crystalline metal oxide^18^. The crystallinity of the ZrO_2_ thin film changed to amorphous upon the addition of Al_2_O_3_. The peak at 28° 2θ° was assigned to the silicon (111)^22^. The solution-processed 2:1 ZAO thin film showed amorphous behavior to 600 °C and even to 700 °C^23^. The amorphous state is more favorable for gate dielectric applications due to the smooth surface and low leakage current. Therefore, the mixed state of gate dielectric with ZrO_2_ and Al_2_O_3_ affects not only the thin-film properties, but also the semiconductor properties.

**Figure 2:**
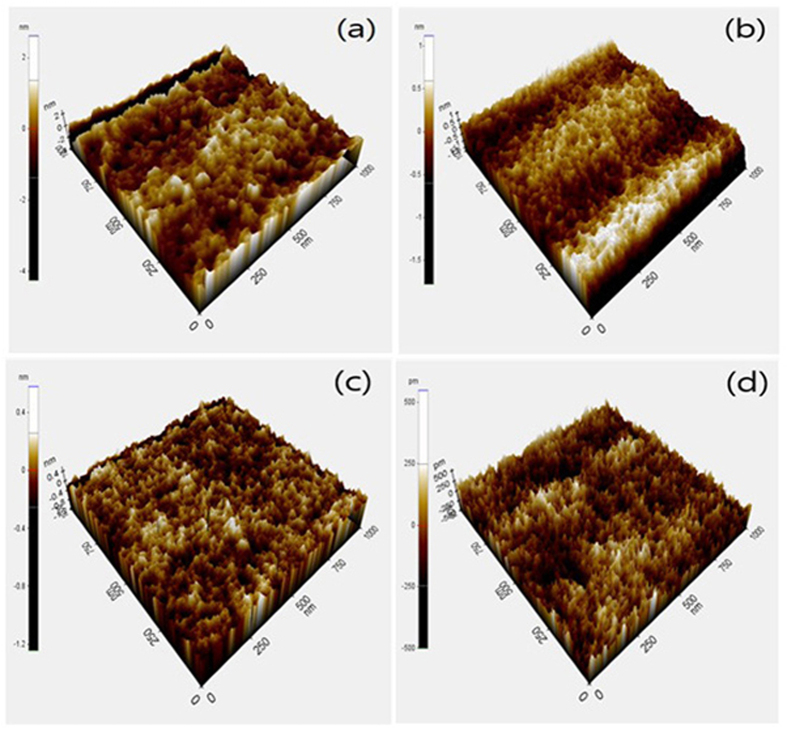
AFM images of ZAO dielectric thin films with a Zr:Al mole ratio of 1:0, 1:1, 1:2, and 1:0 after annealing. Area of 1 μm × 1 μm.

The thicknesses of the ZAO gate dielectrics measured by spectroscopic ellipsometry for mixed mole ratios of ZAO 1:0, 2:1, 1:1, 1:2, and 0:1 was 54, 57, 59, 60, and 63 nm, respectively. In general, an increased gate dielectric thickness results in a lower leakage current; however, a thicker gate dielectric tends to decrease the capacitance. Therefore, the thickness should be controlled properly. The surface roughness of the solution-processed ZAO thin films was measured by AFM, as shown in Fig. 2. The root-mean-square (r.m.s.) roughness of the ZAO dielectric at a mole ratio of 1:0, 1:1, 1:2, and 0:1 of ZAO was 0.697, 0.305, 0.131, and 0.126 nm, respectively. All the dielectrics showed a relatively uniform surface of less than 1 nm of r.m.s. roughness. The rough surface of 1:0 ZAO was related to the crystalline structure of the ZrO_2_ film as confirmed by TGA/DSC and XRD. Small grain-like textures in ZAO (1:0) AFM image were observed, which decreased with the addition of Al_2_O_3_, resulting in improved surface roughness due to the amorphous nature and slow solvent evaporation from the films. The smooth surface of the gate dielectric is important because it reduces the carrier scattering centers, improves the semiconductor/dielectric interface, and achieves good electronic properties^21^.

**Figure 3:**
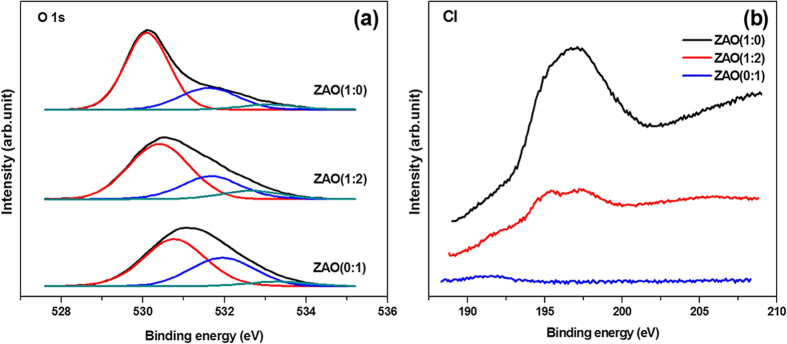
XPS spectra of (**a**) deconvoluted O 1 s core shell with ZAO mole ratio and (**b**) Cl 2p core shell.

XPS analyses of Al, Zr, O, Cl, and N was performed to explore the chemical composition of ZAO gate dielectric thin films, as shown in Fig. 3 and Fig. S2 in the SI. The ZAO thin film containing Zr in the Zr (IV) oxide showed peaks of Zr 3 d_5/2_ at 181.8 eV and Zr 3 d_3/2_ at 184.2 eV with a spin-orbital split of 2.4 eV^4^. The chemical compositions of Zr and Al in the ZAO thin films was similar to the composition of the ZAO precursor solution. The proportions of Zr and Al in the ZAO (1:2) thin film were approximately 35% Zr in the ZAO (1:0) film and approximately 72% Al in the ZAO (0:1) film. The Zr 3 d_5/2_ peak related to the Zr-O bond shifted from 181.8 to 181.9 eV when ZrO_2_ was mixed with Al_2_O_3_, indicating that the Zr-O bonds in ZrO_2_ became more ionic^24^. The XPS spectra of the O 1s core shell was deconvoluted by Gaussian distribution into three peaks, 530.4, 531.6, and 532.6 eV, as shown in Fig. 3(a). The peak centered at 530.4 eV represents the oxygen ions (O^2−^) combined with a metal cation in the ZAO thin films and the peaks at 531.6 eV and 532,6 eV displayed the relationship to the O^2−^ ions located in the oxygen-vacancy regions and boned oxygen such as O_2_, OH, or H_2_O, respectively^17^. The chlorine ions combined with metal ions or substituted into oxygen vacancy in the oxide films could reduce the oxygen vacancy density^25^. In this respect, a chlorine impurity in the ZAO thin films, which comes from the Zr precursors, increased with increasing Zr content as shown in Fig. 3(b), which can bind to metal ions instead of oxygen to produce one electron, resulting in an increase in the leakage current and a decrease in the oxygen vacancies, as in Fig. 3(b) and Fig. S3(d–f) in the SI. The nitrogen in the aluminum precursor was decomposed completely during the process, which was not observed by XPS (Fig. S2 in the SI).

**Figure 4:**
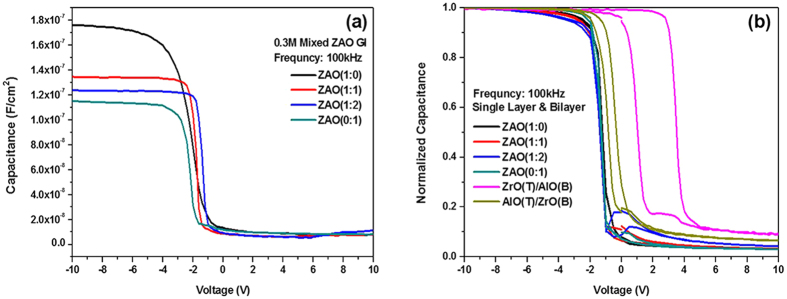
(**a**)Typical C-V characteristics as a function of Zr and Al contents for Al metal/mixed ZAO/p-type Si (Al/ZAO/Si) MOS capacitors and (**b**) hysteresis characteristics of the normalized C-V curves for the same samples shown in figure (**a**) and for MOS capacitors with stacked ZAO bilayer dielectrics at 100 kHz.

To evaluate the quality of the mixed ZAO single layer films and the stacked ZAO bilayer (ZrO/Al_2_O_3_ or Al_2_O_3_/ZrO) films, their capacitance-voltage (C_0_-V) characteristics were measured using Al metal/oxide/p-type Si MOS structures at various frequencies, ranging from 100 kHz to 1 MHz. Figure 4(a) and Table 1 show the C_0_-V curves as a function of the Zr and Al contents at a frequency of 100 kHz for the MOS structures with ZAO.

As the voltage at the Al metal becomes more negative, holes accumulate on the the p-type Si surface. Therefore, the MOS structure operates in the accumulation region and the C_0_-V curves are dominated by the gate-oxide capacitance per unit area (C_ox_), as given by:


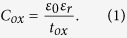


where *ε*_0_ and *ε*_*r*_ are the vacuum permittivity and the dielectric constant of the gate dielectric, respectively, and *t*_*ox*_ is the thickness of the dielectric. Figure 4(a) indicates that the C_ox_ at V = −10 V in the strong accumulation region decreases with increasing Al content. The *ε*_*r*_ values were estimated using the result in Fig. 4(a) and Eq. (1), and they are listed in Table 1 along with other properties, such as C_ox_ and the leakage current density at 5 V. As listed in Table 1, the capacitance and electrical properties were varied with the Zr and Al compositions in the ZAO dielectric films. These results suggest that an increase in the Al contents of the ZAO single layer films causes a decrease in *ε*_*r*_. All the ZAO dielectrics prepared from the solution process showed low capacitance at high frequency operation.

The leakage current of the gate dielectric was increased by the addition of Al_2_O_3_ in the ZrO_2_ matrix because the crystalline lattice structure of ZrO_2_ was changed to a sparse lattice structure (or an amorphous) by Al_2_O_3_, as observed by TGA/DSC and XRD. The proportion of Zr in the ZAO dielectric caused a relatively negative effect of the dielectric on the electrical properties. Recently, it was reported that the capacitance and the leakage current increased for the Zr doped in the Al_2_O_3_ gate dielectric^15^. Additional mobile ions generated by the excess substitution of Zr^4+^ into the Al^3+^ sites in the ZAO thin film caused an unstable frequency-dependent dielectric property^15^. In this experiment, as the portion of Zr in the ZAO thin film decreases, the dielectric properties are degraded, resulting in a lower capacitance and a higher leakage current.

Figure 4(b) also presents the hysteresis characteristics of the normalized C_0_-V curves as a function of the Zr and Al contents at a frequency of 100 kHz for the MOS structures with the ZAO single layer and stacked ZrO_2_-Al_2_O_3_ (ZAO) bilayer. The MOS structures with a ZAO single layer exhibited much smaller distortion and hysteresis in C_0_-V than those of the MOS structures with stacked ZAO bilayer. Figure 4(b) also showed that for the ZAO single layer films prepared with a higher Al content, a more rapid transition from accumulation to inversion occurred with small changes in voltage and the hysteresis was reduced further. These results suggest that a much smaller number of positive defect charge states are present within the ZAO layer and at the Si/ZAO interface for the ZAO films with a higher Al content. In general, the positive defect charges within the insulator and at the semiconductor-oxide interface induces an equivalent negative charge in the semiconductor, which causes a slower transition between a high capacitance and a low capacitance^26^.

**Figure 5:**
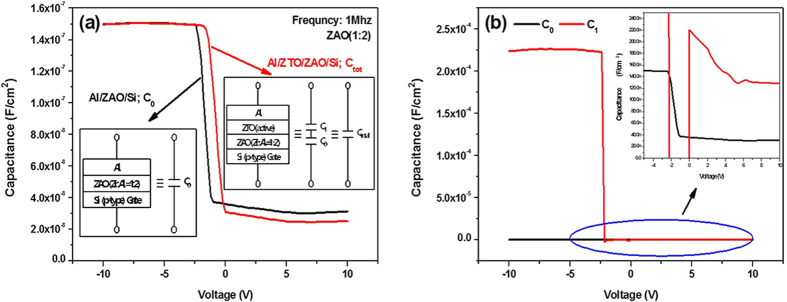
(**a**) Typical C_0_-V and C_tot_-V curves at a frequency of 1 MHz for Al/ZAO(Zr:Al = 1:2)/Si MOS structures and Al/ZTO/ZAO(Zr:Al = 1:2)/Si TFT structures, respectively. The equivalent circuits for both structures are shown in the inset of figure (**a**); (**b**) C_1_-V curves of a ZTO active layer extracted by using the results in figure (a) and Eq. (2). The C_0_-V characteristic is inserted for comparison.

To understand effectively the electrical properties of the developed ZTO-based TFTs with the ZAO gate dielectircs, the capacitance of a ZTO active layer was extracted by assuming that a ZTO active capacitance C_1_ is added in series with C_0_, which is the capacitance of the Al metal/ZAO/p-type Si MOS structures (see Fig. 4(a)), and the total capacitance of Al metal/ZTO/ZAO/p-type Si structures (C_Tot_) is expressed as


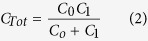


where *C*_*0*_ and *C*_1_ are the capacitance of the Al/ZAO/p-type Si MOS structures and a ZTO active layer, respectively. Figure 5(a) shows the measured C_0_-V and C_tot_-V characteristics at a frequency of 1 MHz for the Al/ZAO/p-type Si MOS structures and Al/ZTO/ZAO/p-type Si TFT structures, respectively, which have a ZAO dielectric with Zr:Al = 1:2. The C-V data from the same structure on n-type Si substrate were also shown in Fig. S3 in the SI. The inset in Fig. 5(a) shows the equivalent circuit for both structures. Figure 5(b) shows the C_1_-V characteristics of the ZTO active layer extracted using the results shown in Fig. 5(a) and Eq. (2). As the voltage applied to the Al metal electrode becomes more negative than −2.4 V, the ZTO surface near the ZAO dielectric is accumulated by electrons. Therefore, for V < −2.4 V, the TFT structure operates in the accumulation region and the much smaller C_0_ compared to C_1_ is dominant in the total capacitance C_Tot_, which makes the C_0_ equivalent to a gate-oxide capacitance C_ox_ expressed by Eq. (1). On the other hand, as shown in Fig. 5(b), the TFT structure operates in the depletion region after V > 0 V, even though its depletion mode needs a settle-down region in the range of −2.4 V < V < 0 V. Negative capacitance was observed in this settle-down region, which is attributed to the inductance-like behavior from a capacitor due to the transition from a depleted capacitor to an accumulated conductor in ZTO active thin films^27^. Because the capacitance in the depletion region for the Al/ZTO/ZAO/Si TFT structures is voltage dependent as shown in Fig. 5, the depletion charge *Q*_*d*_ can be determined using the expression, *Q*_*d*_ = ∫*CdV*. Therefore, based on the results shown in Fig. 5(b), it was estimated that *Q*_*d*_ within the ZTO depletion region induced at V = 8 V was 2.586 × 10^−7^ C/cm^2^.

**Figure 6:**
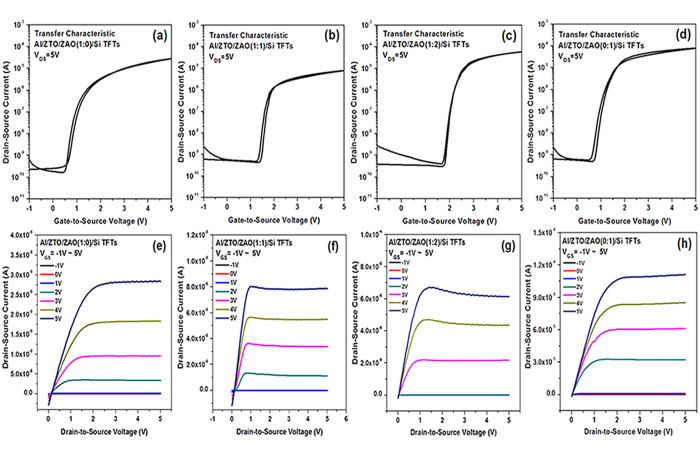
Typical saturation transfer characteristics of ZTO TFTs with a Zr:Al mole ratio of (**a**) 1:0, (**b**) 1:1, (**c**) 1:2, and (**d**) 0:1 measured at V_DS_ of 5 V and the output characteristics as a function of the *V*_*GS*_ for ZTO TFTs with a Zr:Al mole ratio of (**e**) 1:0, (**f**) 1:1, (**g**) 1:2, and (**h**) 0:1.

The transfer and output characteristics of the ZTO TFTs with the ZAO single gate dielectric were measured as a function of the Zr:Al ratio as shown in Fig. 6. The transfer characteristics operating in the saturation region were measured at a fixed drain-to-source voltage (V_DS_) of 5 V. As shown in Fig. 6(a–d), for the TFTs with a higher Al content in ZAO films, better transfer curves with weaker hysteresis characteristics were observed, which is because the smaller defect charge states are present within ZAO for the ZAO films with a higher Al content. It is also noteworthy to mention that the onset voltage (V_ON_), which is defined as the gate-to-source voltage (V_GS_) at which the mobile electron carriers begin to accumulate in the channel and the drain-to-source current (I_DS_) begins to increase in a transfer curve, shifted to the right with increasing Al content in the ZAO films. This suggests that with increasing Al content in the ZAO films, more Al atoms are introduced into ZTO thin films and increase the number of acceptors due to the substitution of Sn sites with Al atoms at the ZTO-ZAO interface, which in turn causes a shift in V_ON_ to the right. The SIMS results in Fig. S4 in the SI confirmed that within the ZTO active layer, the intensity of the Al signal increased with increasing Al content in the ZAO films and the intensity of the Sn signal was one-order of magnitude higher than that of the Zn and O signals. The atomic radii of Al, Sn, Zn, and O when in tetrahedral covalent bonds are 1.26, 1.4, 1.31, and 0.66 Å, respectively^28^. This suggests that the substitution of large Sn sites by small Al atoms occurs readily at the ZTO-ZAO interface.

The output characteristics with mixed ZAO gate dielectrics, as shown in Fig. 6(f,g), showed that the I_DS_ in the saturation region decreases slightly with increasing amount of Al in the ZAO dielectric films, V_GS_, and V_DS_, indicating that electron flow in the channel region becomes slower due not only to the increased number of scattering events in the metallic pathway with a higher Al density at the ZTO-ZAO interface, but also to the enhancement of the potential strength by V_GS_ in the saturation mode. The negative slope was also attributed to the slow traps near the semiconductor–insulator interface, which was considered when deriving the ideal model. The filled slow traps reduce the number of free carriers, resulting in a diminishing current^29^. The output curves also exhibit current-crowding characteristics, suggesting the existence of contact resistance between the source/drain electrode and the ZTO channel. Therefore, this contact resistance should be improved to realize TFTs with the best performance.

The slopes of the sub-threshold swing (*SS*) were obtained from the inverse slopes of the transfer curves in Fig. 6(a–d). The saturation field-effect mobility *μ*_*eff*_ was also estimated using Eq. (3):


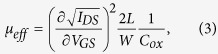


where C_ox_ is the capacitance per unit area of the ZAO gate dielectric listed in Table 1. Table 2 summarizes the important device parameters of the ZTO-based TFTs with mixed ZAO dielectric films, which were obtained from the results shown in Fig. 6(a–d) using Eq. (3). As listed in Table 2, the TFTs with a Zr:Al ratio of 1:2 showed the best device parameters, which included a maximum *μ*_*eff*_ ((*μ*_*eff*_)_*max*_) of 37.01, threshold voltage (*V*_*th*_) of 2 V, on-to-off current ratio of 1.46 × 10^5^, and subthreshold slope (*SS*) of 0.1 V/dec. Although the electrical properties of the TFTs with Al_2_O_3_ based gate dielectric are superior to those of ZTO TFT with ZrO_2_ dielectric, the TFTs with a certain amount of ZrO_2_ in the Al_2_O_3_ gate dielectric showed the best electrical properties. As evident in Table 2, increasing Zr in the ZAO thin film may not be very positive to realize the high performance TFTs because of the film morphology and a chlorine impurity from the Zr precursors as mentioned previously. Compared to the Zr-doped aluminum oxide gate dielectric, the incorporation of one third ZrO_2_ to Al_2_O_3_ can enable good interfacial properties with the ZTO semiconducting layer, resulting in improved properties. As expected, the ZTO TFTs based on the ZAO gate dielectric showed a much lower operating voltage of approximately 5 V, which was attributed to the sol-gel processed high k gate dielectric layer with a low thickness, and better subthreshold slope of 0.1 V/dec compared to a-Si TFT because of the high k value of the dielectric.

The *Q*_*d*_ within the ZTO depletion region was reported to induce a positive shift of *V*_*th*_ (*ΔV*_*th*_) which is expressed as 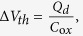 where C_ox_ is given by Eq. (1)^30^. The calculated *ΔV*_*th*_ of 2.1 V, which is in good agreement with *V*_*th*_ of 2 V in Table 2, was obtained using the *Q*_*d*_ estimated from Fig. 5(b) and the C_ox_ listed in Table 2. This confirms that the C_1_-V characteristics extracted from the equivalent circuit model are reasonable.

In the case of stacked gate dielectrics in the ZTO TFTs, a mobility, an on-to-off current ratio, a threshold voltage, and a subthreshold slope of the ZTO TFTs with Al_2_O_3_ (top)/ZrO_2_ (bottom) gate dielectric were 6.51 cm^2^/Vs, 4.49 × 10^4^, 1.99 V, and 0.12 V/dec, whereas those of the ZrO_2_ (top)/Al_2_O_3_ (bottom) dielectric layer showed lower values, as shown in Table S1 and Fig. S5 in the SI. The top layer of the gate dielectrics will play a key role in the formation of a major channel in the active layer for the top contact TFTs. The interface between the top and underneath gate dielectric also contains many trap sites and defects, which deteriorate the electrical properties of the transistors due to carrier scattering and trappings. The change of off-current state with decreasing gate voltage are different for the two stacked gate dielectric-based TFTs. This is because the band gap of ZrO_2_ is 5.8 eV, while that of Al_2_O_3_ is 8.8 eV, which means that carrier injection into ZrO_2_ is easier than Al_2_O_3_^16^. Therefore, the flat off-current region can be seen in the ZrO_2_/Al_2_O_3_ gate dielectric.

**Figure 7:**
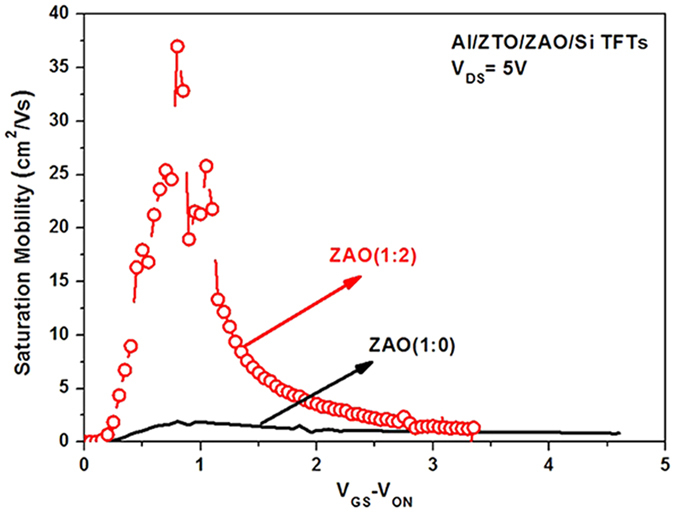
Saturation mobilities (*μ*_*eff*_) at room temperature as a function of *V*_*GS*_ *-V*_*ON*_ estimated from the transfer curves for ZTO TFTs with a Zr:Al mole ratio of 1:0 and 1:2 shown inFig. 6(a,c) by using Eq. (3).

The *V*_*GS*_-*V*_*ON*_ dependence of the *μ*_*eff*_ values estimated from the saturation transfer characteristics plotted in Fig. 6(a–d) using Eq. (3) is shown in Fig. 7 for the a-ZTO TFTs with Zr:Al ratios of 1:0 and 1:2. Here, *V*_*GS*_-*V*_*ON*_ rather than *V*_*GS*_ was used to remove the effect of the trapped charges from *V*_*GS*_, which makes it certain that the saturation mobility at various *V*_*GS*_ values is estimated correctly^31^. As illustrated in Fig. 7, the saturation mobilities at room temperature have a maximum of 37.01 and 1.93 cm^2^/Vs at *V*_*GS*_-*V*_*ON*_ = 0.8 V for a-ZTO TFTs with a Zr:Al ratio of 1:2 and 1:0, respectively, suggesting that the TFTs with a higher Al content in the ZAO dielectric films exhibit higher *μ*_*eff*_ values. A larger number of Al atoms in the ZAO films cause a lower C_ox_, as shown in Table 1 and Fig. 4(a), which results in the introduction of more Al into the ZTO films as shown in the SIMS data. This produces a larger drain current due to a higher conductivity of Al (3.65 × 10^5^ S/cm) than Sn (0.91 × 10^5^ S/cm) and Zn (1.69 × 10^5^ S/cm)^28^. Therefore, a lower C_ox_ and a higher drain current due to the larger number of Al atoms in the ZAO dielectric films in turn induce higher *μ*_*eff*_ values, as expressed in Eq. (3).

## Conclusion

Solution-processed double gate dielectrics were fabricated with ZrO_2_ and Al_2_O_3_ in the form of mixed and stacked types for oxide TFTs. Double coated dielectric films were prepared to produce solid dielectric films. The crystallinity of the ZrO_2_ thin film disappeared and the surface roughness was improved with the Al_2_O_3_ combination. XPS and SIMS analyses confirmed the chemical composition of the thin films of gate dielectrics. A capacitance model was setup for an analysis of the MIS structure. Oxide TFTs with mixed gate dielectrics showed better electrical properties over the TFTs with stacked dielectrics. The best electrical properties of the ZTO TFTs with the ZAO (1:2) gate dielectric showed a field effect mobility of 37.01 cm^2^/Vs, a threshold voltage of 2.00 V, an on-to-off current ratio of 1.46 × 10^5^, and a subthreshold slope of 0.10 V/dec. The capacitance evolution of the semiconductor from the TFT model structure showed a lower C_ox_ and a higher drain current because the larger number of Al atoms in the ZAO films induce higher *μ*_*eff*_ values. These properties were because the amorphous ZAO with low surface roughness and defects induced low trap states at the ZAO-ZTO interface and a larger number of Al atoms in the ZAO films caused a lower C_ox_, a higher drain current, and a higher *μ*_*eff*_ value. The capacitance evolution of the semiconductor from the TFT model structure described well the threshold voltage shift observed in the ZTO TFT with the ZAO (1:2) gate dielectric.

## Methods

Gate dielectric materials for the solution process were prepared from a mixture of zirconyl chloride octahydrate (ZrOCl_2_·8H_2_O, Aldrich) and aluminum nitrate nonahydrate (Al(NO_3_)_3_·9H_2_O, Aldrich) dissolved in 2-methoxyethanol. 0.3 M zirconium aluminum oxide (ZAO) solutions were formulated at zirconium and aluminum ratios of 1:0, 2:1, 1:1, 1:2, and 0:1. As a precursor of oxide semiconductor, 0.3 M ZTO was prepared with a mixture of zinc acetate dihydrate (Zn(CH_3_COO)_2_·2H_2_O, Aldrich) and tin chloride (SnCl_2_, Aldrich) in 2-methoxyethanol with an acetylaceton stabilizer.

To measure the electrical property of ZAO dielectric films, various mole ratios of ZAO were spin coated on a p-type Si wafer. Al/ZAO/p-type Si and Al/ZTO/ZAO/p-type Si wafers were prepared as metal-insulator-semiconductors (MISs). A p-type Si wafer was UV treated for 10 min before the dielectric coating. A ZAO thin film was spin coated at 2000 rpm for 30 sec and pre-baked on a hot plate at 230 °C for 1 hr. for solvent removal and surface alignment, and then annealed 500 °C for 1 hr. In order to make sufficient thickness of 60 nm, the spin coating process was performed twice. ZTO was spin coated as an active layer and then annealed 500 °C for 1 hr. Aluminum was deposited by thermal evaporation to make a rectangular area of 0.25 mm^2^ for the capacitance-voltage measurements. Bottom-gate and top-contact ZTO TFTs were prepared with the same ZAO gate dielectric layer and aluminum (100 nm) to have channel width and length of 1500 μm, and 100 μm, respectively.

The thermal properties of the mixture of ZAO precursors were measured by TGA-DSC (SDT Q600, TA Instruments) under a N_2_ atmosphere. The film thickness was measured by ellipsometry (Elli-SE, Ellipso Technology). X-ray diffraction (XRD; XRD-6100, Shimadzu) was used to identify the crystal structures of the ZAO films coated on the bare Si wafers. XRD was performed using the thin film diffraction technique, in which the samples were fixed at a low angle of 3° to the X-ray beam during the 2θ scan of the detector. The surface characteristics and chemical composition were analyzed by atomic force microscopy (AFM; XE-70, Park Systems Corp.), X-ray photoelectron spectroscopy (XPS; Sigma Probe, Thermo VG ESCA) with surface sputtering and dynamic second ion mass spectroscopy (IMS 4FE7, Cameca Co., SIMS) with a Cs^+^ ion gun were performed. The capacitance-voltage properties of the ZAO gate dielectric in the ZAO-based MIS and the ZTO/ZAO-based TFT were measured using LCR meter (Agilent 4285A). The electrical characteristics of the ZTO TFTs were measured in air and in the dark using a semiconductor parameter analyzer (Keithley 4200). The measurements were typically performed using a continuous method, and the transfer curve was recorded before the output curve.

## Additional Information

**How to cite this article**: Kim, H. *et al*. A mixed solution-processed gate dielectric for zinc-tin oxide thin-film transistor and its MIS capacitance. *Sci. Rep.* **6**, 33576; doi: 10.1038/srep33576 (2016).

## Supplementary Material

Supplementary Information

## Figures and Tables

**Table 1:** Summary of electrical properties of MOS structures with mixed ZAO single layer and stacked ZrO_2_-Al_2_O_3_ bilayer.

Prepared Samples	Thickness [nm]	Gate-oxide capacitance per unit area at 100 kHz [nF/cm^2^]	Estimated dielectric constant at 100 kHz	Leakage current density at 5 V [A/cm^2^]
Mixed ZAO (Zr:Al = 1:0)	54.5	176.1	10.8	5.0 × 10^−7^
Mixed ZAO (Zr:Al = 1:1)	59.4	135.1	9.07	5.9 × 10^−6^
Mixed ZAO (Zr:Al = 1:2)	59.5	123.3	8.30	4.8 × 10^−6^
Mixed ZAO (Zr:Al = 0:1)	63.3	115.4	8.25	1.5 × 10^−7^
Stacked ZrO_2_(T)/Al_2_O_3_(B)	110.8	86.2	10.8	8.9 × 10^−9^
Stacked Al_2_O_3_(T)/ZrO_2_(B)	107.2	154.8	18.8	2.4 × 10^−7^

^*^(T): Top layer, (B): Bottom layer.

**Table 2:** Summary of important device parameters of the ZTO based TFTs with mixed ZAO gate dielectric films.

Mixed ZAO mole ratio	SS (V/dec)	V_ON_ (V)	μ_eff_^a^ (cm^2^/Vs)	V_th_ (V)	On/Off Ratio
Zr:Al = 1:0	0.13	0.4	1.93	0.76	1.68 × 10^5^
Zr:Al = 1:1	0.13	1.2	8.98	1.50	1.61 × 10^4^
Zr:Al = 1:2	0.10	1.65	37.01	2.00	1.46 × 10^5^
Zr:Al = 0:1	0.14	0.4	36.63	1.00	1.36 × 10^5^

^a^It represents a maximum value of *μ*_*eff*_ and depends on the value of *V*_*GS*_ *– V*_*ON*_.
