# Supplementary material for: A mixed solution-processed gate dielectric for zinc-tin oxide thin-film transistor and its MIS capacitance
Source: Sci Rep. 2016 Sep 19;6:33576. doi: 10.1038/srep33576 (PMC5027534; doi:10.1038/srep33576)
Supplement: Supplementary Information [file srep33576-s1.pdf]

Supplemental Information

A mixed solution-processed gate dielectric for zinc-tin oxide thin-film transistor and its MIS capacitance

Hunho Kim[1], Young-Jin Kwack[1], Eui-Jung Yun[2] and Woon-Seop Choi[1]*

[1]Department of Display Engineering, Hoseo University,

[2]Department of ICT and Automotive Engineering, Hoseo University

Asan, 31499, Korea

* CORESSPONDING AUTHOR: Woon-Seop Choi (E-Mail: wschoi@hoseo.edu)

Table S1 Summary of electrical properties of the ZTO TFTs with stacked ZAO gate dielectrics

Stacked ZAO	μ_{sat} (cm^2/Vs)	On/Off Ratio	V_{th} (V)	SS (V/dec)
ZrO(T)/AlO(B)	0.78	1.25×10^5	0.73	0.29
AlO(T)/ZrO(B)	6.51	4.49×10^4	1.99	0.12

* (T): Top layer, (B): Bottom layer

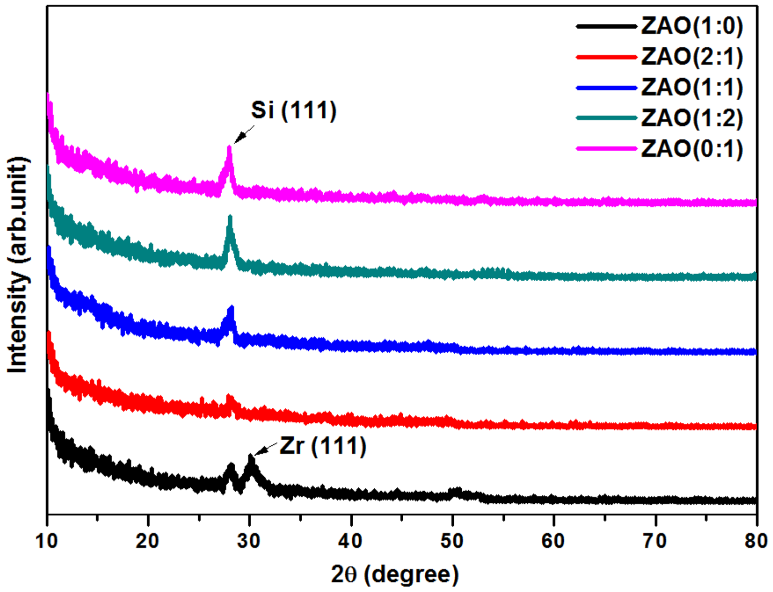

Fig. S1 XRD spectra of ZAO thin films with a Zr:Al mole ratio of 1:0, 2:1, 1:1, 1:2, and 1:0.

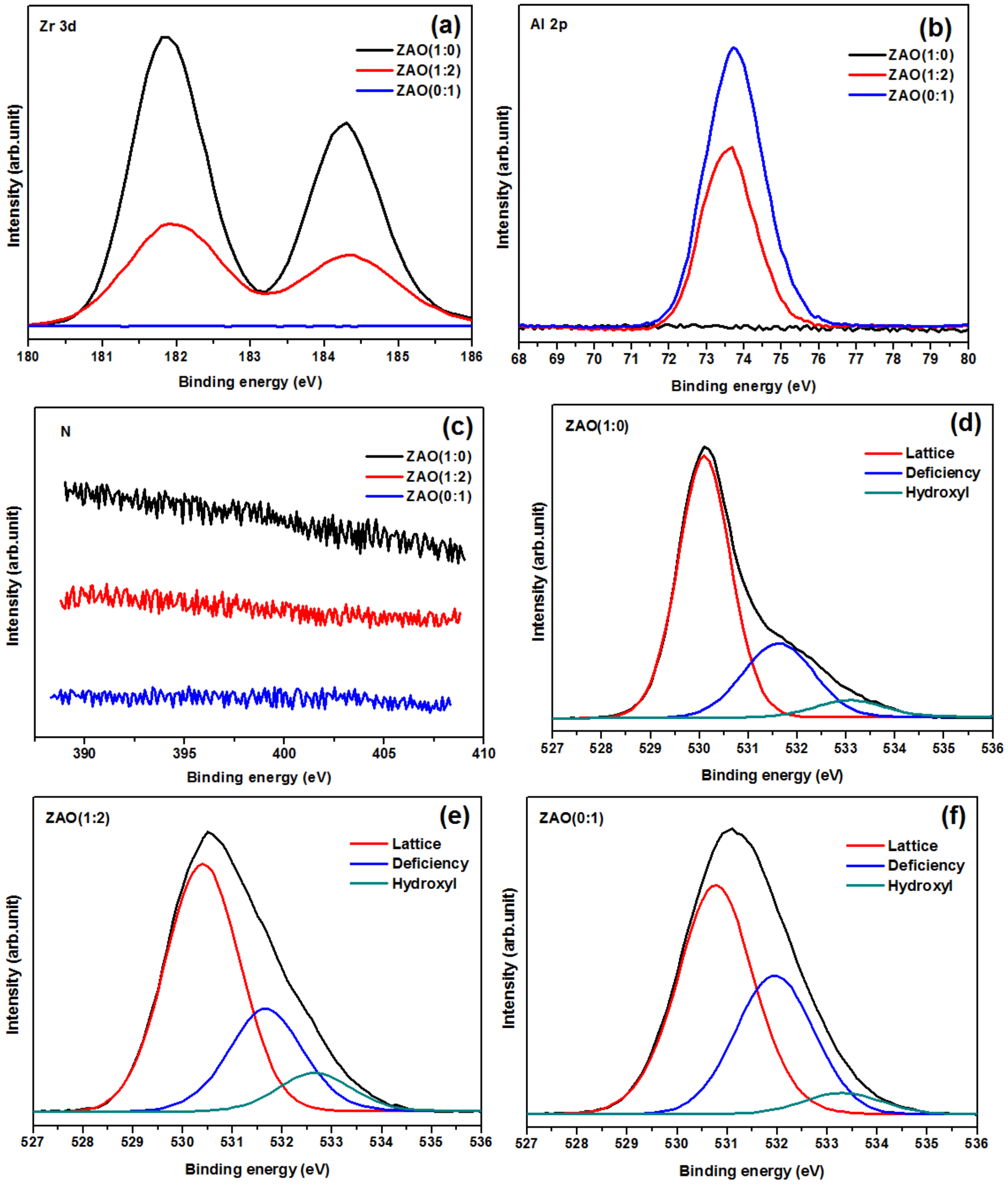

Zr 3d
(a)
ZAO(1:0)
ZAO(1:2)
ZAO(0:1)
Intensity (arb.unit)
Binding energy (eV)
Al 2p
(b)
ZAO(1:0)
ZAO(1:2)
ZAO(0:1)
Intensity (arb.unit)
Binding energy (eV)
N
(c)
ZAO(1:0)
ZAO(1:2)
ZAO(0:1)
Intensity (arb.unit)
Binding energy (eV)
ZAO(1:0)
(d)
Lattice
Deficiency
Hydroxyl
Intensity (arb.unit)
Binding energy (eV)
ZAO(1:2)
(e)
Lattice
Deficiency
Hydroxyl
Intensity (arb.unit)
Binding energy (eV)
ZAO(0:1)
(f)
Lattice
Deficiency
Hydroxyl
Intensity (arb.unit)
Binding energy (eV)

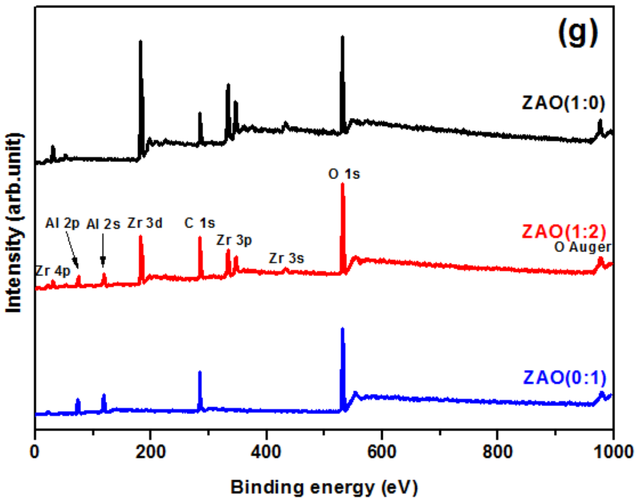

Fig. S2 XPS spectra of various atoms of Al, Zr, O, Cl and N in the ZAO dielectric thin films after thermal and sputtering treatment

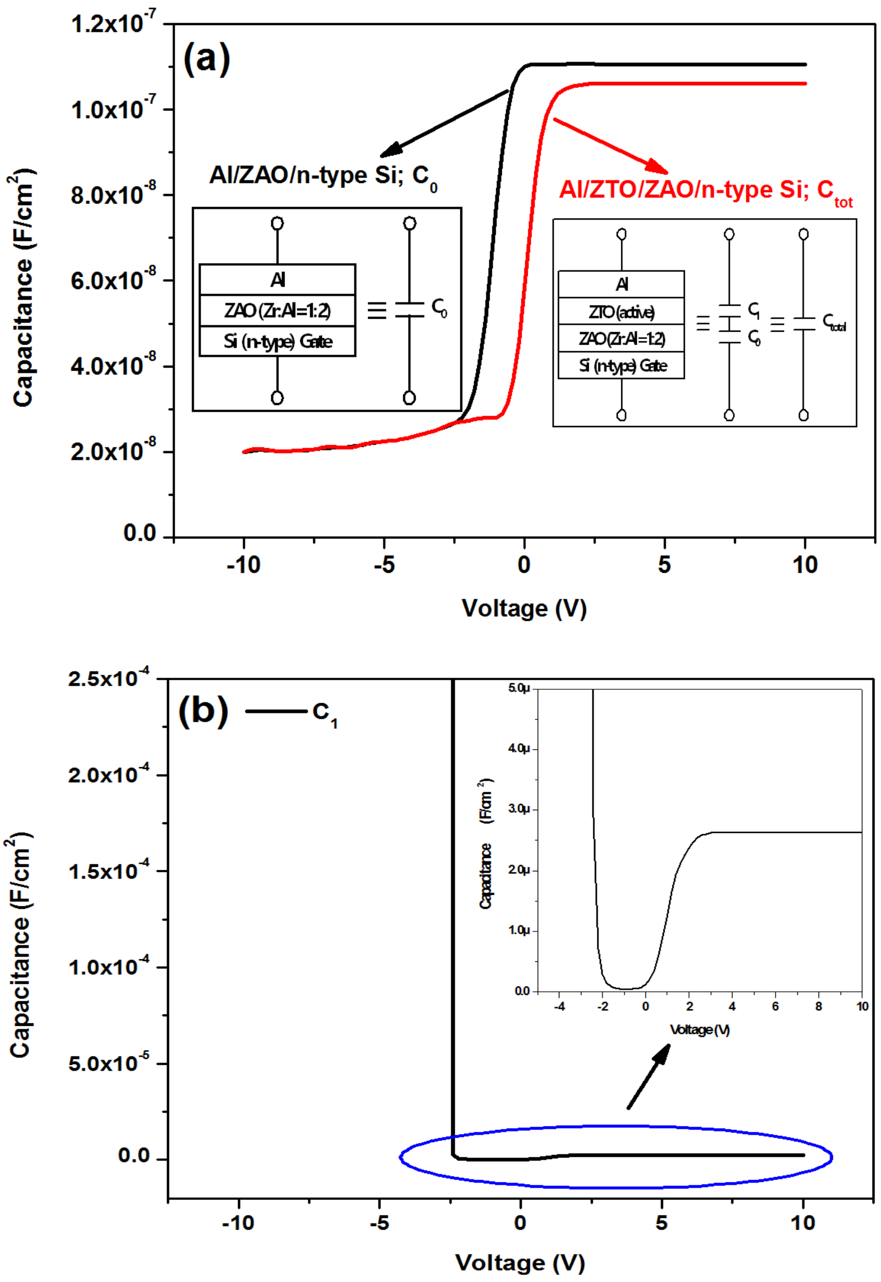

Fig. S3 (a) Typical C_0-V and C_{tot}-V curves at a frequency of 1 MHz for Al/ZAO(Zr :Al = 1:2)/n-Si MOS structures and Al/ZTO/ZAO(Zr :Al = 1:2)/n-Si TFT structures, respectively. (b) C_1-V curves of a ZTO active layer extracted by using the results in figure (a) and Eq. (2).

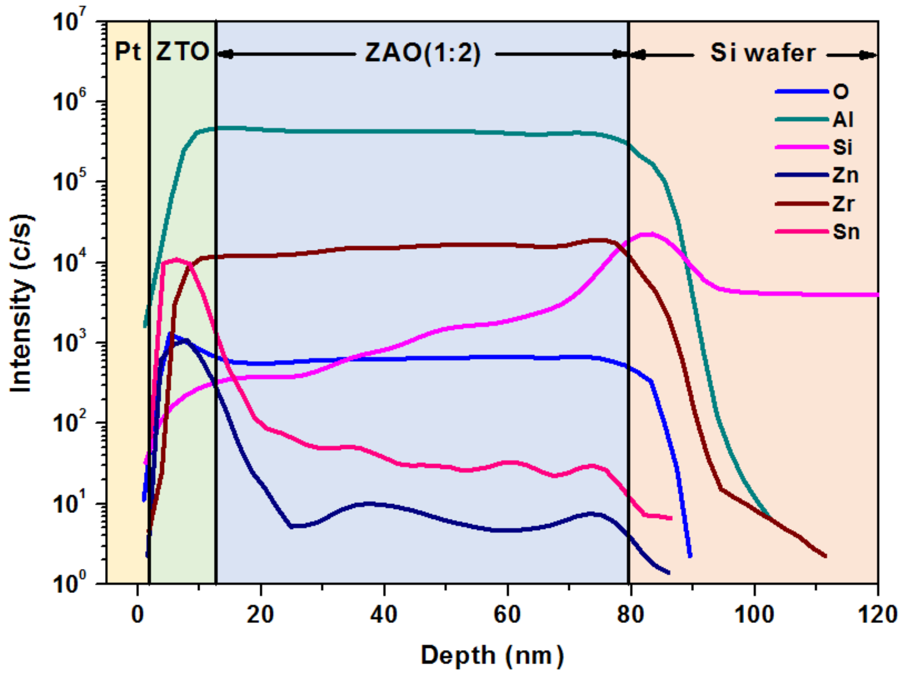

Fig. S4 SIMS spectra of Al/ZTO/ZAO/p-doped Si TFT structure in depth profile with sputtering.

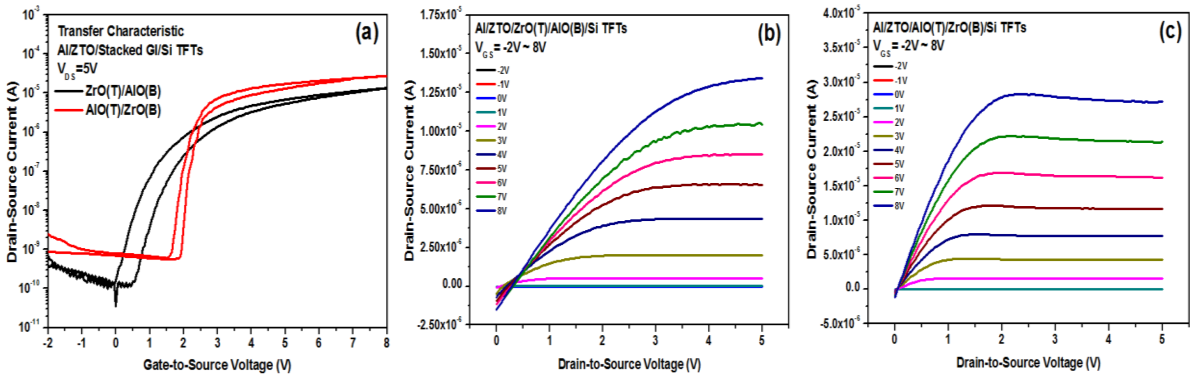

Fig. S5 Typical saturation transfer characteristics of ZTO TFTs with stacked ZAO gate dielectrics (a) transfer curves measured at V_{DS} of 5V, (b) output curves of ZrO_2(T)/Al_2O_3(B), and (c) output curves of Al_2O_3(T)/ZrO_2(B) as a function of the V_{GS} for ZTO TFTs.
